# Replacing Potassium in the Emergency Department May Not Decrease the Hospital Mortality in Mild Hypokalemia: A Propensity Score Matching Analysis

**DOI:** 10.3390/medicina59111912

**Published:** 2023-10-29

**Authors:** Wachira Wongtanasarasin, Nattikarn Meelarp

**Affiliations:** 1 Department of Emergency Medicine, Faculty of Medicine, Chiang Mai University, Chiang Mai 50200, Thailand; nattikarn.m@cmu.ac.th; 2 Department of Emergency Medicine, UC Davis School of Medicine, Sacramento, CA 95817, USA

**Keywords:** potassium, mild hypokalemia, emergency department, propensity score, mortality

## Abstract

*Background*: Hypokalemia is associated with considerable morbidity and mortality, highlighting the timely correction of potassium levels as a critical medical consideration. However, the management of mild hypokalemia remains a subject of ongoing debate. This study explores the relationship between potassium replacement in the emergency department (ED) and hospital mortality in patients with mild hypokalemia. *Methods*: This retrospective cohort study was conducted at a tertiary care hospital, including patients who presented to the ED with mild hypokalemia, defined as potassium levels between 3.0 and 3.4 mmol/L, between 2020 and 2021. Patients diagnosed with acute coronary syndrome, diabetic ketoacidosis, hyperglycemic hyperosmolar state, and major cardiac arrhythmias were excluded. The patient cohort was then divided into two groups, based on whether they received potassium replacement in the ED. A propensity score analysis was employed to account for potential pretreatment confounding factors, including age, gender, time on ED arrival, insurance, comorbidities, serum potassium and creatinine levels, and ED length of stay. Subsequently, a multivariable logistic regression analysis, incorporating hospital length of stay and acute comorbidities, was performed post-matching to further adjust for predictive factors. The primary outcome was all-cause hospital mortality. *Results*: This study included a total of 1931 patients, of which 724 were matched for analysis (362 with potassium replacement and 362 without). The average age was 53.9 years, and most were male (58.5%). After adjusting for confounding factors using propensity score analysis, there was no significant difference in hospital mortality between the potassium replacement and control groups (adjusted odds ratio 0.81, 95% CI 0.36–1.79, *p* = 0.60). *Conclusions*: This study’s findings indicate that replacing potassium in the ED may not lower the risk of hospital mortality in patients with mild hypokalemia. Consequently, the customary practice of potassium replacement in hospitalized patients may lack justification, and deferring the replacement until after patients leave the ED could be considered.

## 1. Introduction

Potassium is one of the crucial ions within the body, with significant implications for its functioning [1,2,3]. Most (98%) of potassium is found intracellularly [2]. However, even minor changes in the extracellular potassium level can profoundly impact cardiovascular function, potentially leading to cardiac arrhythmias and sudden cardiovascular death [1,2,3]. Imbalances in potassium levels pose significant challenges in the field of emergency medicine. Previous studies reported that approximately 5.5–11% of patients presenting at the emergency department (ED) have hypokalemia, defined as serum potassium below 3.5 mmol/L [4,5]. Furthermore, Vuillaume et al. found that hypokalemia is frequently observed in the ED and was related to worse outcomes [6].

While severe hypokalemia is associated with specific electrocardiographic changes and cardiac arrhythmias, patients with mild hypokalemia (serum potassium 3.0–3.4 mmol/L) are typically asymptomatic and may not require aggressive treatments [4,7]. Hypokalemia has been linked to an increased risk of cardiac arrhythmias in individuals with acute coronary syndrome (ACS) [8]. However, no evidence currently confirms that correcting hypokalemia decreases the likelihood of clinical cardiac arrhythmias and death. To our knowledge, no evidence exists regarding the risks and benefits of restoring serum potassium levels in hypokalemic patients in the ED.

Given that hypokalemia is a common observation, even in patients without ACS, and is associated with significant costs and utilization of hospital resources [4], it is imperative to investigate the potential consequences in these populations. Correcting mild hypokalemia may be considered an unnecessary procedure that could be postponed until patients are discharged from the ED. Therefore, this study aims to explore the association between potassium replacement at the ED and in-hospital all-cause mortality in patients with mild hypokalemia. We hypothesize that patients with mild hypokalemia at the ED do not require potassium replacement, as it is not associated with hospital mortality.

## 2. Materials and Methods

### 2.1. Study Design and Setting

This retrospective observational study was conducted at a single tertiary hospital comprising 25 ED and 1500 in-hospital beds. Data were extracted from electronic medical records. While there were no official guidelines for electrolyte replacement during the study period, serum potassium levels were commonly measured in the ED for various reasons. All methods were performed following the Declaration of Helsinki. The study protocol received approval from the institutional review board, Faculty of Medicine, Chiang Mai University. It was granted exemption from written informed consent due to its retrospective design and minimal risk (IRB No. EME-8946/2022). Patient confidentiality was preserved by using anonymous health records. This research was conducted and reported following the guidelines outlined in the Strengthening the reporting of observational studies in epidemiology (STROBE) statement [9].

### 2.2. Study Participants

The study included patients who presented to the ED between January 2020 and December 2021 and had recorded serum potassium level measurements. Mild hypokalemia was defined as a serum potassium level ranging from 3.0 to 3.4 mmol/L. Exclusion criteria included patients who had serum potassium levels outside the specific range, individuals aged 18 years or younger, and those with acute coronary syndrome, diabetic ketoacidosis, hyperglycemic hyperosmolar state, and major cardiac arrhythmia (including atrial fibrillation, atrial flutter, supraventricular tachycardia, ventricular tachycardia, ventricular fibrillation, and Mobitz type II second-degree or third-degree atrioventricular block).

These exclusion criteria were implemented to ensure a focus on patients where serum potassium levels may impact disease management [10,11,12]. If multiple serum potassium measurements were obtained during a single ED visit, only the initial measurement was considered for inclusion in the study. Participants were divided into two groups based on their potassium replacement status at the ED, with potassium replacement defined as administering any form of potassium (intravenous or oral). It is important to note that other treatments were determined on a case-by-case basis depending on individual patient status and diagnosis at the ED. Additionally, there was no standardized protocol for monitoring potassium levels after potassium replacement.

### 2.3. Data Collection and Outcome Measures

A systematic search of electronic medical records was conducted to identify eligible patients meeting the predefined criteria. Baseline demographic information, clinical features, and potassium replacement status during the ED stay were collected. Information regarding hospital characteristics for included patients was also documented. All variables were predetermined and detailed in a coding guide for abstractors. The primary outcome measure of interest was in-hospital all-cause mortality.

### 2.4. Statistical Analysis

Data were analyzed using Stata version 16.1 (StataCorp., College Station, TX, USA). Statistical significance was defined as a two-tailed *p*-value less than 0.05. Descriptive statistics were used to summarize baseline characteristics and hospital information. Categorical data are presented as frequencies and percentages, while continuous data are expressed as the means and standard deviations or medians and interquartile ranges, as appropriate. The standardized difference (STD) was utilized to assess differences in each variable between the two groups. An absolute STD value of less than 10% indicated no significant difference between the groups and suggested minimal potential confounding by indication at baseline. This measure facilitated quantifying and comparing the magnitude of differences across various factors under consideration [13].

Given the non-randomized nature of our study, which aimed to evaluate the prognosis of different treatments, there was a possibility of selection bias and imbalances in prognostic factors. To mitigate these biases, propensity scores were employed to match the two groups based on potassium replacement status. These methods are recognized for observational studies that lack randomization [13,14]. Propensity scores, derived from multiple logistic regression, estimated the probability of an individual receiving potassium replacement. The model incorporated variables such as age, gender, time of ED arrival, insurance status, comorbidities, serum potassium levels, serum creatinine levels, and ED length of stay. The propensity scores were then divided into ten blocks with similar probabilities of receiving potassium in the ED. Patients receiving potassium replacement were matched with those, in a one-to-one ratio, within each block. Following matching, the balance of baseline characteristics between the two groups was assessed using STD, to ascertain the successful minimization of differences and ensure comparability.

Subsequently, a multiple logistic regression, adjusted for hospital characteristics including hospital length of stay and acute comorbidities, was applied to compare the proportion of in-hospital mortality.

## 3. Results

**Figure 1:**
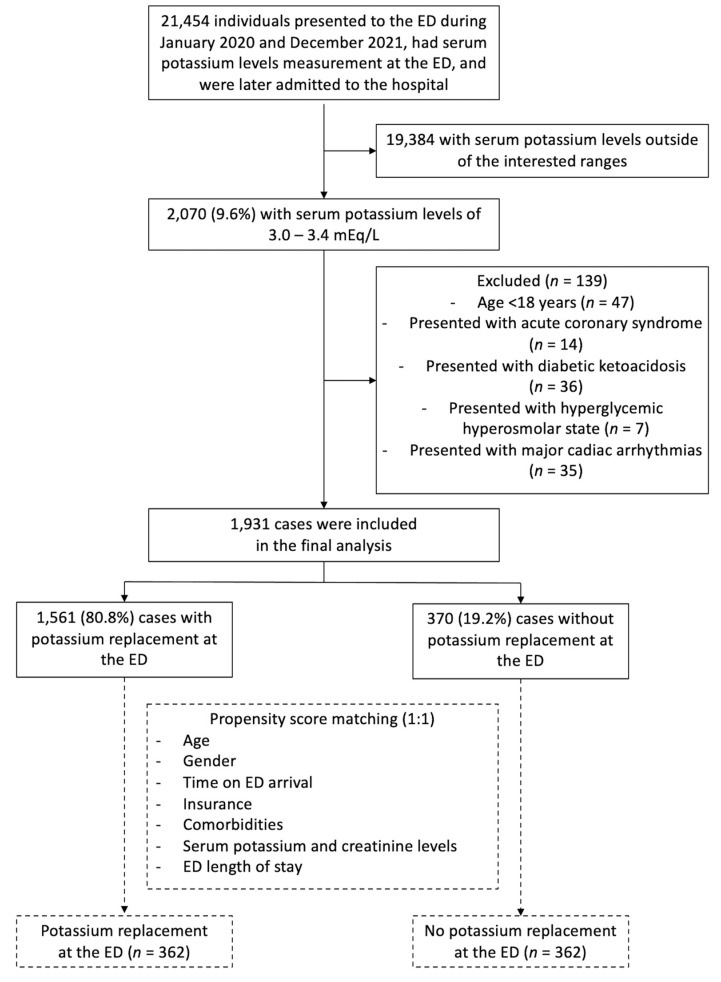
Study flow diagram of the patient cohort. Abbreviations: ED, emergency department.

A total of 21,454 individuals who presented to the ED during the study period were eligible, of whom 2070 (9.6%) had mild hypokalemia. From this group, 139 were excluded based on predefined criteria: 47 were under the age of 18, 14 were diagnosed with ACS, 36 were diagnosed with diabetic ketoacidosis, 7 were diagnosed with hyperglycemic hyperosmolar state, and 35 presented with major cardiac arrhythmias (Figure 1).

The final analysis included 1931 patients, of whom 1561 received potassium replacement at the ED and 370 did not. Baseline and hospital characteristics, as well as propensity scores, are summarized in Table 1. Significant differences were observed in age, insurance status, presence of diabetes, chronic heart failure, serum potassium levels, and ED length of stay, with standardized differences exceeding 10% for all variables.

**Figure 2:**
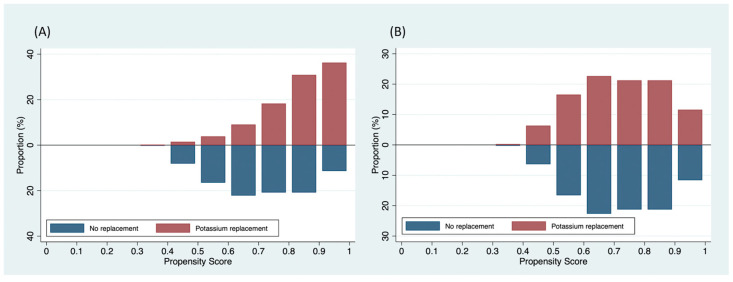
The distribution of the propensity scores between the two groups: (**A**) original cohort and (**B**) propensity score-matched cohort.

These differences suggested potential imbalances between the groups in terms of these variables. To address this, one-to-one propensity score matching was performed, resulting in a final sample of 724 patients, equally divided between the potassium replacement group and the no replacement group (362:362) (Figure 2). The propensity score model used for matching is provided in Table 2. Table 1 also demonstrates the baseline and hospital characteristics of the propensity score-matched cohorts. All imbalance variables were found to be reasonably similar, as indicated by a standardized difference of less than 10%.

There was no statistical significance between patients in the replacement group and those without, regarding the primary outcome (Table 3). After adjusting for hospital length of stay and acute morbidities, patients who received potassium replacement at the ED had 0.81 odds of having the outcome (95% CI 0.36–1.79, *p* = 0.60), compared to those who did not.

## 4. Discussion

Our study demonstrates that approximately 10% of individuals who presented to the ED and had serum potassium level measurements were found to have mild hypokalemia. Potassium replacement at the ED was not significantly associated with the risk of hospital mortality when employing propensity score analysis and controlling for other potential confounders. Interestingly, our findings indicated that up to 80% of patients with mild hypokalemia received treatment at the ED through intravenous or oral routes.

The results of our study are consistent with previous research conducted in Korea and Switzerland, which reported hypokalemia rates of 5.5% and 11%, respectively, emphasizing the significance of this issue in the ED [4,5]. Previous studies demonstrated that hypokalemia (serum potassium levels < 3.5 mmol/L) was associated with increased odds of mortality during hospital admission [4,6,15]. The American College of Cardiology/American Heart Association guidelines recommend promptly correcting serum potassium levels in patients with severe hypokalemia or in those with potassium levels below 3.0 mmol/L who present with cardiac arrhythmias or hypokalemia-related symptoms [16]. However, the optimal management of mild hypokalemia remains controversial. In clinical practice, this mild form has raised questions about the necessity and timing of potassium replacement. Most research in the ED has predominantly focused on specific populations with certain medical conditions, such as ACS [17,18] and acute heart failure [19]. These studies have delved into the management of electrolyte imbalances, but often in high-risk subsets. In contrast, the general population presenting at the ED, which constitutes a broader and more diverse patient demographic, has received less attention in the context of potassium disturbances and their clinical implications. On the other hand, our study serves as a piece of evidence, including those in the ED without high-risk or specific management, regarding potassium replacement in hypokalemic patients.

Similar to our findings, some studies have suggested that routine potassium replacement in patients with mild hypokalemia may not be necessary and could potentially lead to adverse events such as hyperkalemia, especially in patients with comorbidities such as renal disease [20]. It is a widespread practice to correct a patient’s potassium level in the situation of any hypokalemia, because of the perceived threat of cardiac arrhythmia [3]. Hypokalemia was found to be associated with an increased incidence of atrial fibrillation in those with acute ST-elevation myocardial infarction or undergoing percutaneous coronary intervention [8,10,21]. However, this treatment could be unpleasant for patients, due to the great amount and unpleasant taste of potassium pills or any inconvenience associated with intravenous infusion [22]. Furthermore, correcting mild hypokalemia, especially in the ED, also increases the number of blood samples and hospitalization costs while not influencing patient outcomes [23]. Previous literature by Harkness et al. documented that correcting serum potassium levels to ≥3.5 mmol/L did not benefit hypokalemic patients without ACS or a history of cardiac arrhythmias during hospitalization [7]. Our study further supports these findings by demonstrating that individuals with mild hypokalemia presenting to the ED and subsequently admitted to the hospital face a similar risk of hospital mortality, regardless of potassium replacement treatment at the ED, suggesting that this common clinical practice may be unwarranted [3,7,8,10,21,22,23].

Several limitations should be acknowledged in our study. First, it was conducted at a single tertiary care hospital, potentially limiting the generalizability of the findings. Second, the study’s retrospective design introduces the possibility of missing or inaccurately recorded variables, which may introduce bias. Third, we did not differentiate between patients with mild hypokalemia due to different underlying causes, which could have impacted the results. Furthermore, while we excluded patients with potentially confounding factors (i.e., ACS, diabetic ketoacidosis, and major cardiac arrhythmias), it was not possible to eliminate all risk factors that might influence the outcome. In addition, this study only focused on all-cause hospital mortality, which may limit the interpretation of the results. It is imperative to assess the impact of potassium replacement on cardiovascular mortality. Finally, there needed to be a standardized protocol for managing these patients. The route and amount of potassium replacement depended on the clinician’s decisions, which could affect the outcomes. It would be beneficial to replicate this study with a larger sample size, and a prospective study design may be necessary to address issues related to documentation and limitations associated with retrospective reviews.

## 5. Conclusions

In conclusion, our study provides evidence that potassium replacement in the ED may not reduce the risk of all-cause hospital mortality in patients with mild hypokalemia. These findings suggest that the routine practice of potassium replacement in hospitalized patients may be unnecessary, and potassium replacement could be deferred until after patients are discharged from the ED. However, further research is warranted to confirm these findings and determine the optimal management approach for mild hypokalemia in the ED.

## Figures and Tables

**Table 1:** Characteristics and potential confounders of the included participants.

Characteristics	Original Cohort					Propensity-Matched Cohort				
	Replacement (*n* = 1561)		No Replacement (*n* = 370)		STD	Replacement (*n* = 362)		No Replacement (*n* = 362)		STD
	*n*	(%)	*n*	(%)		*n*	(%)	*n*	(%)	
**Baseline characteristics**										
Age (years), mean ± SD	55.6	±20.0	46.0	±20.1	+0.478	46.8	±20.1	46.3	±20.1	+0.026
Male gender	899	57.6	228	61.6	−0.082	223	61.6	226	62.4	−0.017
Time on ED arrival					−0.020					+0.003
08:01–16:00	348	22.3	122	33.0		112	30.9	117	32.3	
16:01–24:00	597	38.2	122	33.0		129	35.6	120	33.2	
00:01–08:00	616	39.5	126	34.0		121	33.4	125	34.5	
Insurance					−0.141					−0.028
Universal coverage scheme	673	43.1	163	44.1		170	47.0	158	43.7	
Government officer	317	20.3	53	14.3		57	15.8	53	14.6	
Social security scheme	144	9.2	41	11.1		42	11.6	39	10.8	
Cash payment	368	23.6	94	25.4		67	18.5	94	26.0	
Stateless people	59	3.8	19	5.1		26	7.2	18	5.0	
Comorbidities										
Diabetes	190	12.2	31	8.4	+0.125	28	7.7	30	8.3	−0.020
Chronic heart failure	10	0.6	5	1.4	+0.476	8	2.2	4	1.1	+0.087
Chronic kidney disease	144	9.2	25	6.8	+0.091	28	7.7	24	6.6	+0.043
Route of potassium replacement					N/A					N/A
Oral	1253	80.3	0	0		257	71.0	0	0	
Intravenous	738	47.3	0	0		180	49.7	0	0	
Serum potassium level (mmol/L), mean ± SD	3.23	±0.14	3.31	±0.12	−0.607	3.30	±0.11	3.31	±0.12	−0.070
Serum creatinine level (mg/L), median (IQR)	0.86	(0.68–1.16)	0.87	(0.69–1.09)	+0.027	0.85	(0.68–1.11)	0.87	(0.68–1.09)	+0.011
ED length of stay (hours), median (IQR)	3.8	(2.4–5.4)	3.0	(2.0–4.7)	+0.353	2.8	(1.9–4.4)	3.0	(2.1–4.5)	−0.033
**Hospital Characteristics**										
Hospital length of stay (days), median (IQR)	7	(4–15)	4	(2–8)	+0.445	7	(4–14)	4	(2–8)	+0.464
Hospital length of stay					+0.118					+0.186
<7 days	875	56.1	225	60.8		192	53.0	220	60.8	
7–30 days	583	37.4	129	34.9		141	39.0	126	34.8	
>30 days	103	6.6	16	4.3		29	8.0	16	4.4	
Acute comorbidities										
Sepsis	57	3.7	10	2.7	+0.054	17	4.7	10	2.8	+0.102
Acute heart failure	74	4.7	11	3.0	+0.092	16	4.4	10	2.8	+0.089
Propensity score, mean ± SD	0.83	±0.12	0.72	±0.15	+0.872	0.72	±0.14	0.72	±0.14	+0.041

Abbreviations: ED, emergency department; IQR, interquartile range; N/A, not applicable; SD, standard deviation; STD, standardized difference.

**Table 2:** Derivation of propensity scores via a multivariable logistic regression model.

Equation Parameters	Coefficient	Standard Error	95% Confidence Interval	*p*-Value
Age (year)	0.022	0.003	0.015, 0.029	<0.001
Male gender	0.079	0.130	−0.175, 0.334	0.542
Time on ED arrival	0.207	0.078	0.055, 0.359	0.008
Insurance	−0.049	0.034	−0.117, −0.019	0.160
Comorbidities				
Diabetes	0.048	0.236	−0.414, 0.509	0.840
Chronic heart failure	−1.364	0.603	−2.547, −0.182	0.024
Chronic kidney disease	−0.016	0.312	−0.628, 0.596	0.959
Serum potassium level (mEq/L), mean ± SD	−0.525	0.053	−0.630, −0.421	<0.001
Serum creatinine level (mg/L), median (IQR)	−0.080	0.061	−0.199, 0.040	0.192
ED length of stay (hours), median (IQR)	0.135	0.320	0.073, 0.198	<0.001

Abbreviations: ED, emergency department; IQR, interquartile range; SD, standard deviation.

**Table 3:** Study outcome.

Clinical Outcome	Replacement (*n* = 362)		No Replacement (*n* = 362)		Treatment Effect (Potassium Replacement vs. No Replacement at ED)						
	*n*	%	*n*	%	Parameter	Unadjusted Analysis			Adjusted Analysis ^†^		
						Effect	95% CI	*p*-Value	Effect	95% CI	*p*-Value
All-cause hospital mortality	12	3.3	14	3.9	Odds ratio	0.85	0.39, 1.87	0.69	0.81	0.36, 1.79	0.60

Abbreviations: CI, confidence interval; ED, emergency department. ^†^ Multivariable logistic regression analysis adjusted for hospital characteristics (hospital length of stay, and acute comorbidities, including sepsis and acute heart failure).

## Data Availability

Not applicable.
